# Automatic Movement Recognition for Evaluating the Gross Motor Development of Infants

**DOI:** 10.3390/children12030310

**Published:** 2025-02-28

**Authors:** Yin-Zhang Yang, Jia-An Tsai, Ya-Lan Yu, Mary Hsin-Ju Ko, Hung-Yi Chiou, Tun-Wen Pai, Hui-Ju Chen

**Affiliations:** 1 Department of Computer Science and Information Engineering, National Taipei University of Technology, Taipei 10608, Taiwan; t111598063@ntut.edu.tw (Y.-Z.Y.); t111c71004@ntut.edu.tw (J.-A.T.); 2 Division of Pediatric Neurology, Department of Pediatrics, MacKay Children Hospital, Taipei 104217, Taiwan; yalan.6211@mmh.org.tw; 3 Department of Pediatric Neurology, Hsinchu Municipal MacKay Children Hospital, Hsinchu 300195, Taiwan; mk.2112@mmh.org.tw; 4 Institute of Population Health Science, National Health Research Institute, Miaoli County 350401, Taiwan; hychiou@nhri.edu.tw; 5 School of Public Health, College of Public Health, Taipei Medical University, Taipei City 110301, Taiwan; 6 Department of Computer Science and Engineering, National Taiwan Ocean University, Keelung 20224, Taiwan; 7 Department of Medicine, Mackay Medical College, New Taipei City 25200, Taiwan

**Keywords:** developmental delay, pose estimation, transfer learning, machine learning

## Abstract

Objective: The objective of this study was to early-detect gross motor abnormalities through video detection in Taiwanese infants aged 2–6 months. Background: The current diagnosis of infant developmental delays primarily relies on clinical examinations. However, during clinical visits, infants may show atypical behaviors due to unfamiliar environments, which might not truly reflect their true developmental status. Methods: This study utilized videos of infants recorded in their home environments. Two pediatric neurologists manually annotated these clips to identify whether an infant possessed the characteristics of gross motor delays through an assessment of his/her gross motor movements. Using transfer learning techniques, four pose recognition models, including ViTPose, HRNet, DARK, and UDP, were applied to the infant gross motor dataset. Four machine learning classification models, including random forest, support vector machine, logistic regression, and XGBoost, were used to predict the developmental status of infants. Results: The experimental results of pose estimation and tracking indicate that the ViTPose model provided the best performance for pose recognition. A total of 227 features related to kinematics, motions, and postures were extracted and calculated. A one-way ANOVA analysis revealed 106 significant features that were retained for constructing prediction models. The results show that a random forest model achieved the best performance with an average F1-score of 0.94, a weighted average AUC of 0.98, and an average accuracy of 94%.

## 1. Introduction

Developmental delay is when children exhibit delays in one or more aspects of development, including physical growth, language communication, cognition, psychosocial skills, or self-care abilities. It is considered a nonspecific neuropsychiatric symptom, and the diagnosis can be categorized into functional and etiological types. A functional diagnosis is typically determined by early intervention specialists based on assessments across various developmental domains. Early intervention involves an integrative approach combining medical rehabilitation, special education, family support, and social welfare services. These interventions could provide timely support to children with developmental delays, especially for infants living in rural areas with a shortage of medical resources. Between 1997 and 2008, the prevalence of developmental delays increased significantly, rising from 0.16% to 3.25%, with an annual growth rate of 20% [1]. According to the Taiwan Social and Family Affairs Administration at the Ministry of Health and Welfare, the reporting rate of children with developmental delays increased from 1.24% in 2010 to 3.01% in 2022. However, it remains significantly lower than the global prevalence rate of 6–8%, as estimated by the World Health Organization. Additionally, a study revealed that the actual prevalence of developmental delays among preschool children in northeastern Taiwan has already reached 11.36% [2]. This finding highlights a significant discrepancy, with the reported rate falling notably short of the actual prevalence. The ages between 0 and 3 years represent a critical period for rapid synaptic growth in the developing brain and serve as a golden period for early therapeutic intervention. During this time period, external stimulation and positive interactions can effectively promote healthy development across various domains in infants and young children [3].

Traditional motion assessment methods often rely on wearable devices to capture data on movement. While these approaches offer high precision, they require direct contact with infants, which might cause discomfort and atypical responses. Moreover, the direct-contact nature of these devices limited their applicability in the context of telemedicine. Recent advancements in deep learning and computer vision have led to the development of video-based, no-contact human pose recognition as an effective alternative for monitoring and analyzing human motion [4]. Studies have shown that machine learning classification methods based on pose recognition features can effectively detect abnormal movement patterns in newborn babies. Using 31 neonatal videos, these methods successfully identified writhing movements on the second and third postnatal days, demonstrating their efficacy in distinguishing abnormal movement patterns [5]. Additionally, using data from 127 videos of preterm infants, a method combined skin model segmentation and large displacement optical flow for motion tracking and extracted kinematic features to classify preterm infant movements as typical or atypical. This method achieved 92% accuracy and successfully predicted the risk of subsequent cerebral palsy [5]. Similarly, an automated general movement assessment used representative learning techniques to distinguish normal and abnormal movement patterns in preterm infants, and achieved an accuracy of 95% [6]. Moreover, using OpenPose, a 25-point skeletal model was extracted from infant videos and was analyzed using a shallow multilayer neural network. This approach effectively distinguished between fidgety and non-fidgety movements, achieving an average classification accuracy of 88% [7]. However, most of these studies focused on the classic Prechtl general movement assessment to predict neurological disorders, such as cerebral play. Some studies posed early gross motor development, and movement abnormalities may suggest later autism or other neurodevelopmental disability development in children [8]. More motor feature pattern analysis may aid the early diagnosis of neurodevelopmental delays.

In collaboration with the Department of Early Evaluation and Treatment Center, Mackay Children Hospital, this study developed an automatic supporting system for assessing gross motor pattern in infants and young children. This system utilizes videos of spontaneous infant movements recorded by parents at home to create a Taiwanese infant pose recognition dataset and a developmental health status dataset. Utilizing pose estimation technologies, the system automatically detects key feature points on the limbs of infants and quantifies movement features to assess their gross motor development. The results are expected to enable timely interventions and provide effective strategies to support infants with developmental challenges, ultimately fostering healthier developmental outcomes for those with motor and learning impairments.

## 2. Materials and Methods

**Figure 1:**
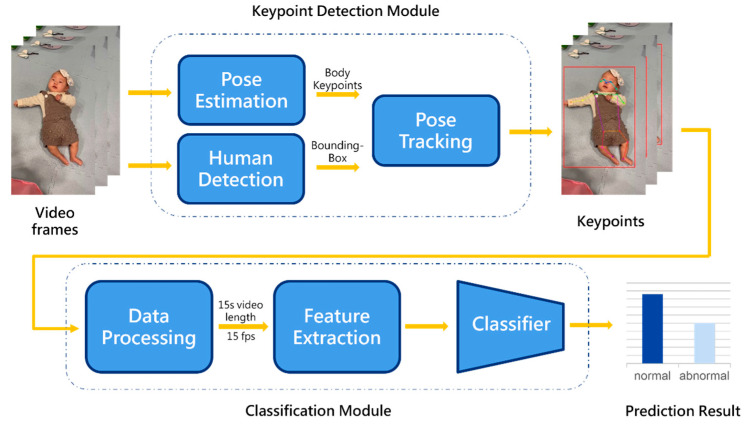
System architecture.

This study used recorded videos to conduct an automated assessment of gross motor movements for infants aged 0–6 months. As shown in Figure 1, the system architecture consists of a keypoint detection and gross motor development classification module, enabling a completely automated analysis process to detect development delays.

### 2.1. Health Dataset on Infant Development

In collaboration with the Department of Early Evaluation and Treatment Center, Mackay Children Hospital (Taiwan) collected spontaneous movement video data from children aged 0–6 months, including typical-development infants and infants with a gross motor development delay. We invited participants through the Online Neurodevelopmental Evaluation (ONE) System, and the study recruitment period during 29 March 2021~1 June 2024. (IRB no: 20MMHIS447e, IRB no: 23MMHIS113e). The collected videos were screened to select video segments that satisfied the specified criteria for the dataset. During video recording, participants were instructed to capture footage while the infant was awake and lying supine on the bed, freely moving their arms and legs. The recording was obtained from directly above the infant to ensure clear visibility of their head, arms, torso, and legs. Selected videos had to maintain proper camera angles, with no obstructions or interferences covering the body of the infant. All videos were recorded at a frame rate of 15 frames per second. Two pediatricians independently classified and cross-reviewed the collected videos. Among these video clips, 26 video segments were identified as developmental delays in those infants.

### 2.2. Infant Gross Motor Dataset

**Figure 2:**
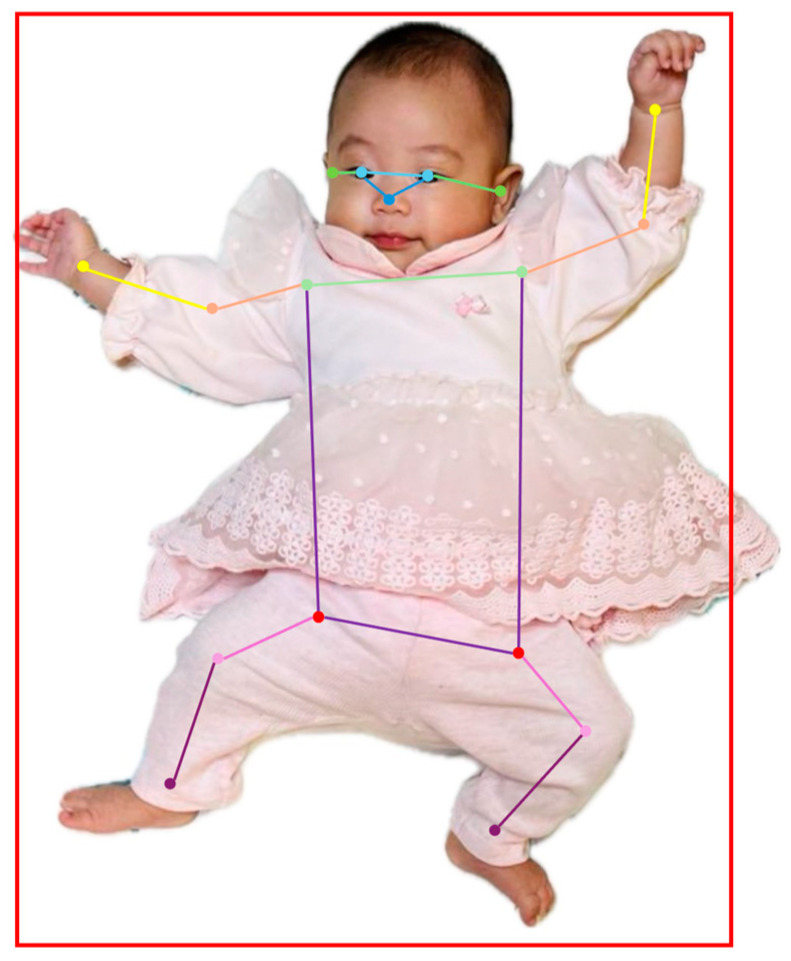
Example of pose estimation annotation. Red bounding box: encompassing the infant’s body; Color lines: movement lines of 17 body joints.

Pose estimation serves as a preliminary task for classifying developmental delays in infants. Commonly used pose estimation datasets, such as the MS COCO (Microsoft Common Objects in Context) dataset [9] and MPII Human Pose dataset (Max-Planck Institute for Informatics) [10], primarily focus on images of adults. However, the significant differences in limb proportions between adults and infants made these datasets unsuitable for this study. Therefore, a new infant-specific dataset was created for training infant pose estimation models. This dataset includes 858 images extracted from the spontaneous movement videos of 69 infants. Each image was annotated using LabelMe(v.4.6.0), following the MSCOCO format, incorporating a bounding box encompassing the body and 17 keypoints, as shown in Figure 2. To expand the dataset, 500 real infant images from the synthetic and real infant pose (SyRIP) dataset [11] were incorporated simultaneously. Combined with the collected images from this study, a total of 1358 annotated images formed the dataset used for this study.

### 2.3. Detection of Keypoints

The study applied four different models for comparison, including ViTPose [12], HRNet [13], DARK [14], and UDP [15] for pose estimation tasks. Among them, both DARK and UDP were based on HRNet-W48. Based on the pre-trained parameters of each model, fine-tuning was performed using the infant gross motor dataset. A stratified cross-validation approach was used, dividing the dataset into five subsets. During training procedures, the bounding boxes for human detection were adjusted to a fixed aspect ratio (4:3), cropped, and then resized to fixed dimensions (256 × 192 or 384 × 288). Data augmentation techniques were applied, including random rotations between [−45°, 45°] and random scaling. The object keypoint similarity metric was used to evaluate the accuracy of human keypoint detection, as shown in Equation (1). Here, *d_i_* represents the Euclidean distance between the *i*th detected keypoint and its ground truth, *s* is the pixel area of the predicted object, and ki is the normalization factor for the *i*th keypoint, calculated based on the MSCOCO dataset. The models were evaluated in terms of mean average precision (mAP), mean average recall (mAR), and inference speed on the testing set. As shown in Equation (2), where *N* is the number of threshold settings, *N* had the following values of 0.50, 0.55, …, 0.90, 0.95. ${AP}_{i}$ and ${AR}_{i}$ denote precision and recall at the *i*th threshold setting, respectively.

(1) $$OKS=\frac{\sum_{i}exp(-d_{i}^{2}/2s^{2}k_{i}^{2})\delta (v_{i}>0)}{\sum_{i}\delta (v_{i}>0)}$$

(2) $$mAP=\frac{1}{N}\sum_{i}^{N}{AP}_{i}\;\;\;mAR=\frac{1}{N}\sum_{i}^{N}{AR}_{i}$$

A top–down approach was applied for pose estimation in this study. YOLOv9-E was applied to automatically detect infant positions [16]. The best-performing pose estimation model was selected for the detected keypoints. Pose tracking was subsequently applied to match the detected keypoints across consecutive frames, generating skeletal sequential sequences for further analysis.

### 2.4. Classification of Gross Motor Development Abilities

**Figure 3:**
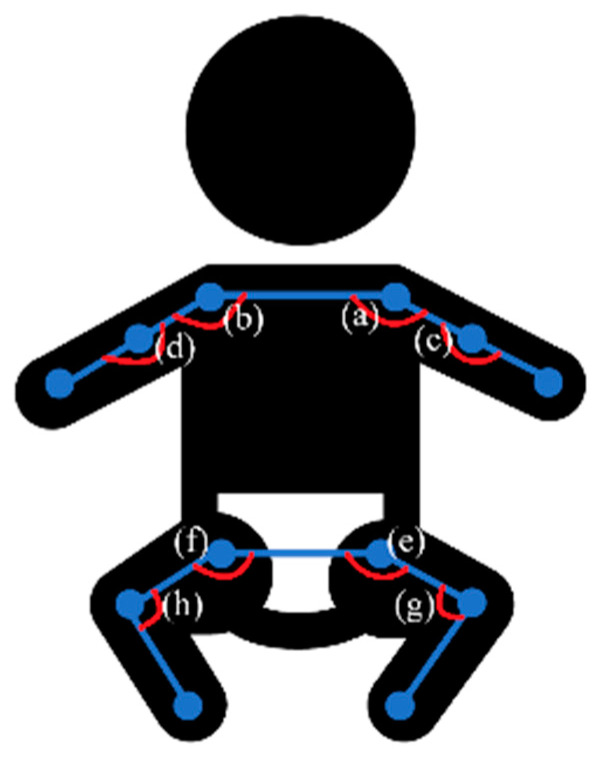
Eight joint angle features of the shoulders (a) and (b), elbows (c) and (d), hips (e) and (f), and knees (g) and (h).

The gross motor development classification module applied skeletal sequential sequences extracted from each video. Keypoints with confidence scores < 0.5 were filtered out, and linear interpolation was applied for the missing values. Since minor discrepancies in pose estimation across frames might result in significant joint jitter in the evaluated videos, a rolling-mean filter with a smoothing window of five frames was applied to temporally smooth the skeletal sequential sequences. The system designated the midpoint of the shoulders as a reference point to account for variations in the distance of the camera setup and infant positioning during recording. The entire skeleton is rotated and normalized based on the calculated trunk length in each frame. This ensures consistent comparison and analysis across different recording conditions. These videos were then segmented into non-overlapping 15 s clips, and the mean joint movement speed was calculated for each segment. The cumulative average movement speed was used to select video clips with a total mean movement speed > 0.9 (normalized trunk length/s). Kinematics, motion, and posture features were calculated and analyzed for these selected clips. The average and maximum movement speed and acceleration of the elbows, wrists, knees, and ankles were extracted. Additionally, eight joint angle features of the shoulders, elbows, hips, and knees are shown in Figure 3. Further analysis included calculating the correlation of the movement speed and acceleration between pairs of joints (e.g., shoulders, elbows, hips, and knees) within a sliding window width of five frames. Features such as the angular velocity and acceleration of elbows, wrists, knees, and ankles, as well as the joint angles, angular velocities, and accelerations of shoulders, elbows, hips, and knees, were extracted comprehensively. Moreover, entropy-based features were also computed for the movement speed and acceleration of elbows, wrists, knees, and ankles.

After feature extraction and calculation, this study utilized four different machine learning models, including Random Forest, Support Vector Machine, Logistic Regression, and XGBoost, to classify the gross motor developmental levels of infants. The dataset consisted of 83 randomly selected non-redundant clips from typically developing infants and 26 clips of infants with developmental delays. To address the imbalance in the dataset, the clips of infants with developmental delays were oversampled to 78 samples. These clips were then standardized for the following classification tasks. To optimize predictive performance, stratified cross-validation was used, and variance analysis was performed on all features. Only features with significant differentiations were used to train and construct the prediction models. This feature-filtering approach ensured the selection of an optimal model for classifying gross motor developmental abilities.

## 3. Results

This study established a dataset comprising videos from 90 infants, resulting in 313 continuous 15 s video segments. We conducted five-fold cross-validation to compare four pose estimation models in terms of architecture, input size, parameter count, processing speed (fps), mAP, and mAR. The detailed performance of different pose estimation approaches is shown in Table 1. The experimental results indicated that ViTPose processes only 139.3 frames per second, whereas HRNet-based models can handle approximately 200 frames per second. Despite the speed differences, ViTPose outperformed the other models in terms of mAP and mAR. All models achieved mAP and mAR scores > 0.9, demonstrating their exceptional performance in the domain of infant pose recognition.

This study used a one-way ANOVA (analysis of variance) to compare the data of infants with typical development and infants with developmental delays, assessing whether individual features exhibited significant differences. A *p*-value < 0.05 was considered statistically significant. Among the original 227 features, only 106 were identified as significantly different features and were considered important features for constructing predictive models for automated developmental ability assessment. Details of the original 227 features and selected 106 features are shown in the Supplementary Materials.

Four machine learning models were used to construct developmental ability assessment prediction models. Each model was constructed and evaluated through a five-fold cross-validation. The models were implemented using scikit-learn (version 1.1.3) and XGBoost (version 2.0.3) and compared using performance metrics, including accuracy, recall, precision, and the F1-score. Table 2 summarizes the performance of the four prediction systems across these mentioned metrics. Our experimental results indicate that the random forest model provided the most stable performance, achieving the highest weighted average scores in accuracy, precision, and the F1-score. Additionally, both XGBoost and logistic regression models performed relatively well with regard to the weighted average recall rates. Metrics with superior performance are highlighted in bold in Table 2.

## 4. Discussion

This study was conducted in collaboration with the Department of Early Evaluation and Treatment Center, Mackay Children Hospital (Taiwan). It utilized spontaneous movement videos collected from infants to develop an automated analysis system for assessing gross motor development in infants. Based on the collected video dataset and automated detection of joint motion features, the system offered a method to observe the limb movements of infants from his/her familiar home environment. Additionally, it could provide physicians with an effective, real-time, remote, and objective mechanism for early assessments of infant developmental delays.

The different pose estimation tools used in this study successfully processed the constructed infant gross motor dataset, achieving over 90% accuracy across the four models. These results indicate a robust performance of our automated detection models in detecting infant limbs under complex environmental conditions and automatically obtaining skeleton sequential sequences for the following analysis on infant developmental delays.

During data preprocessing and feature extraction, 227 features were initially extracted from joint points, including shoulders, elbows, wrists, hips, knees, and ankles, based on the kinematics, motion, and posture characteristics. Through the statistical validation of these 227 features, only 106 features were statistically significant for their predictive capability. In this study, we applied four different machine learning techniques to construct our prediction models. All statistically validated features from the videos of 83 typically developing infants and videos of 26 infants with developmental delays were automatically extracted, trained, and used to construct four different predictive models for comparison. All these four models achieved recall rates > 90%, demonstrating their reliability in correctly identifying most cases of developmental delays.

This automated assessment system addresses the limitations of in-clinic evaluations, where infants may experience anxiety or discomfort in unfamiliar environments, potentially affecting the accuracy of professional assessments. By enabling accurate remote evaluation of the natural activities of infants at home, the system can reliably detect developmental delays through video-based recognition. Furthermore, a cloud-based service platform could allow parents to upload videos of their infant’s movements following standardized filming guidelines. This proposed system could provide quick and accurate assessments of limb movement development at home, offer educational resources, or recommend early diagnosis and treatment whenever necessary. Such a platform enables parents to detect potential symptoms of developmental delays at early stages and ensure timely interventions during critical therapeutic periods, aligning with the overarching goals of smart healthcare.

With the global trend of declining birth rates, increasing attention should be paid to the early detection of developmental delays. In the era of rapid technological advancements, including widespread smartphone usage and cloud services, integrating the automated assessment mechanism proposed in this study could significantly enhance the effective and efficient detection of gross motor development delays in infants, especially for infants residing in rural areas that have a shortage of medical resources. The automated assessment mechanism is a potential advantage in telemedicine. This is particularly relevant for monitoring in remote areas where access to pediatricians or neurologists may be limited.

## Figures and Tables

**Table 1:** Comparison table for pose estimation models.

Method	Backbone	Input Size	Params (M)	Speed (fps)	mAP	mAR
ViTPose	ViTPose-H	256 × 192	632	139.3	0.970	0.976
HRNet	HRNet-W48	384 × 288	64	200.2	0.964	0.969
DARK	HRNet-W48	384 × 288	64	198.5	0.967	0.973
UDP	HRNet-W48	384 × 288	64	200.1	0.967	0.972

**Table 2:** Comparison of performance on developmental delay detection by four different approaches.

Prediction Model	Average Accuracy	Average Recall	Average Precision	Average F1 Score
Random Forest	0.944	0.923	0.965	0.941
XGBoost	0.938	0.962	0.921	0.938
SVM	0.925	0.937	0.919	0.924
Logistic regression	0.913	0.963	0.877	0.914

## Data Availability

The original contributions presented in this study are included in the article/Supplementary Materials. Further inquiries can be directed to the corresponding authors.

## Supplementary Materials

The following supporting information can be downloaded at: https://www.mdpi.com/article/10.3390/children12030310/s1, Supplementary File S1: Original 227 features and selected 106 features (bold face).

## Abbreviations

The following abbreviations are used in this manuscript:

LDOF	large Displacement Optical Flow
GMA	General Movement Assessment
CP	Cerebral Palsy
SMNN	Shallow Multilayer Neural Network
SyRIP	Synthetic and Real Infant Pose
ViTPose	Vision Transformer for Pose Estimation
HRNet	High-Resolution Net
DARK	Distribution-Aware Coordinate Representation of Keypoints
UDP	Unbiased Data Processing
OKS	Object Keypoint Similarity
mAP	Mean Average Precision
mAR	Mean Average Recall
SVM	Support Vector Machine
ANOVA	Analysis of Variance
