# Supplementary material for: Automatic Movement Recognition for Evaluating the Gross Motor Development of Infants
Source: Children (Basel). 2025 Feb 28;12(3):310. doi: 10.3390/children12030310 (PMC11940954; doi:10.3390/children12030310)
Supplement: Supplementary file 1 [file children-12-00310-s001.zip › children-3457340-supplementary.pdf]

Supplementary File S1: Original 227 features and selected 106 features (bold-face).

ID	Feature Name	F-value	P-value
F1	left_elbow_mean_velocity	3.61E-01	5.48E-01
F2	right_elbow_mean_velocity	3.25E-02	8.57E-01
F3	left_wrist_mean_velocity	4.92E-01	4.83E-01
F4	right_wrist_mean_velocity	1.70E+00	1.94E-01
F5	left_knee_mean_velocity	7.69E-01	3.81E-01
F6	right_knee_mean_velocity	1.61E+00	2.06E-01
**F7**	**left_ankle_mean_velocity**	**7.57E+00**	**6.23E-03**
F8	right_ankle_mean_velocity	4.56E-01	5.00E-01
**F9**	**left_elbow_max_velocity**	**5.31E+00**	**2.18E-02**
F10	right_elbow_max_velocity	1.04E+00	3.09E-01
**F11**	**left_wrist_max_velocity**	**1.91E+01**	**1.61E-05**
F12	right_wrist_max_velocity	2.77E-02	8.68E-01
F13	left_knee_max_velocity	4.66E-01	4.95E-01
**F14**	**right_knee_max_velocity**	**1.66E+01**	**5.54E-05**
F15	left_ankle_max_velocity	3.45E+00	6.42E-02
F16	right_ankle_max_velocity	7.29E-01	3.94E-01
F17	left_elbow_mean_acceleration	1.28E+00	2.60E-01
**F18**	**right_elbow_mean_acceleration**	**7.09E+00**	**8.08E-03**
**F19**	**left_wrist_mean_acceleration**	**3.97E+00**	**4.71E-02**
**F20**	**right_wrist_mean_acceleration**	**1.24E+01**	**4.87E-04**
F21	left_knee_mean_acceleration	7.66E-02	7.82E-01
F22	right_knee_mean_acceleration	1.50E+00	2.22E-01
F23	left_ankle_mean_acceleration	2.14E+00	1.44E-01
**F24**	**right_ankle_mean_acceleration**	**7.69E+00**	**5.85E-03**
F25	left_elbow_max_acceleration	1.42E-04	9.90E-01
F26	right_elbow_max_acceleration	1.54E+00	2.15E-01
**F27**	**left_wrist_max_acceleration**	**4.24E+00**	**4.01E-02**
F28	right_wrist_max_acceleration	3.72E-03	9.51E-01
F29	left_knee_max_acceleration	9.76E-01	3.24E-01
F30	right_knee_max_acceleration	2.41E+00	1.21E-01
F31	left_ankle_max_acceleration	2.01E+00	1.57E-01
F32	right_ankle_max_acceleration	1.46E-04	9.90E-01
F33	left_shoulder_mean_angle_velocity	7.94E-03	9.29E-01
F34	right_shoulder_mean_angle_velocity	9.83E-03	9.21E-01
F35	left_elbow_mean_angle_velocity	1.51E-03	9.69E-01
F36	right_elbow_mean_angle_velocity	2.80E+00	9.52E-02
F37	left_hip_mean_angle_velocity	1.18E+00	2.79E-01

F38	right_hip_mean_angle_velocity	2.29E-03	9.62E-01
F39	left_knee_mean_angle_velocity	1.09E+00	2.97E-01
F40	right_knee_mean_angle_velocity	9.63E-03	9.22E-01
F41	left_shoulder_max_angle_velocity	8.97E-03	9.25E-01
F42	right_shoulder_max_angle_velocity	7.53E-02	7.84E-01
**F43**	**left_elbow_max_angle_velocity**	**9.62E+00**	**2.07E-03**
**F44**	**right_elbow_max_angle_velocity**	**1.65E+01**	**6.09E-05**
F45	left_hip_max_angle_velocity	2.14E+00	1.44E-01
F46	right_hip_max_angle_velocity	4.51E-02	8.32E-01
**F47**	**left_knee_max_angle_velocity**	**8.13E+00**	**4.60E-03**
F48	right_knee_max_angle_velocity	3.14E+00	7.72E-02
F49	left_shoulder_mean_angle_acceleration	2.17E+00	1.42E-01
F50	right_shoulder_mean_angle_acceleration	1.41E+00	2.36E-01
**F51**	**left_elbow_mean_angle_acceleration**	**4.21E+00**	**4.08E-02**
**F52**	**right_elbow_mean_angle_acceleration**	**5.84E+00**	**1.62E-02**
F53	left_hip_mean_angle_acceleration	6.18E-02	8.04E-01
F54	right_hip_mean_angle_acceleration	5.12E-01	4.75E-01
F55	left_knee_mean_angle_acceleration	1.34E+00	2.48E-01
F56	right_knee_mean_angle_acceleration	1.43E+00	2.32E-01
F57	left_shoulder_max_angle_acceleration	8.61E-01	3.54E-01
F58	right_shoulder_max_angle_acceleration	1.12E+00	2.91E-01
**F59**	**left_elbow_max_angle_acceleration**	**1.02E+01**	**1.56E-03**
**F60**	**right_elbow_max_angle_acceleration**	**1.19E+01**	**6.16E-04**
**F61**	**left_hip_max_angle_acceleration**	**6.30E+00**	**1.25E-02**
F62	right_hip_max_angle_acceleration	2.86E+00	9.18E-02
**F63**	**left_knee_max_angle_acceleration**	**7.93E+00**	**5.12E-03**
F64	right_knee_max_angle_acceleration	3.35E+00	6.80E-02
**F65**	**left_wrist_velocity_vs_right_wrist_velocity_mean_correlation**	**1.14E+01**	**7.93E-04**
**F66**	**left_wrist_velocity_vs_left_ankle_velocity_mean_correlation**	**6.60E+00**	**1.06E-02**
F67	left_wrist_velocity_vs_right_ankle_velocity_mean_correlation	1.37E-01	7.11E-01
**F68**	**right_wrist_velocity_vs_left_ankle_velocity_mean_correlation**	**3.08E+01**	**5.54E-08**
**F69**	**right_wrist_velocity_vs_right_ankle_velocity_mean_correlation**	**5.22E+01**	**2.94E-12**

**F70**	**left_ankle_velocity_vs_right_ankle_velocity_mean_correlation**	**2.02E+01**	**9.46E-06**
F71	left_wrist_acceleration_vs_right_wrist_acceleration_mean_correlation	1.78E+00	1.83E-01
**F72**	**left_wrist_acceleration_vs_left_ankle_acceleration_mean_correlation**	**8.93E+00**	**3.00E-03**
F73	left_wrist_acceleration_vs_right_ankle_acceleration_mean_correlation	2.67E+00	1.03E-01
**F74**	**right_wrist_acceleration_vs_left_ankle_acceleration_mean_correlation**	**2.95E+01**	**1.04E-07**
**F75**	**right_wrist_acceleration_vs_right_ankle_acceleration_mean_correlation**	**2.90E+01**	**1.29E-07**
**F76**	**left_ankle_acceleration_vs_right_ankle_acceleration_mean_correlation**	**2.08E+01**	**7.01E-06**
**F77**	**left_shoulder_angle_velocity_vs_right_shoulder_angle_velocity_mean_correlation**	**6.03E+00**	**1.46E-02**
F78	left_shoulder_angle_velocity_vs_left_hip_angle_velocity_mean_correlation	1.53E+00	2.17E-01
F79	left_shoulder_angle_velocity_vs_left_elbow_angle_velocity_mean_correlation	5.69E-01	4.51E-01
F80	left_shoulder_angle_velocity_vs_right_elbow_angle_velocity_mean_correlation	3.19E-01	5.73E-01
**F81**	**left_shoulder_angle_velocity_vs_right_hip_angle_velocity_mean_correlation**	**9.07E+00**	**2.78E-03**
**F82**	**left_shoulder_angle_velocity_vs_left_knee_angle_velocity_mean_correlation**	**6.84E+00**	**9.26E-03**
**F83**	**left_shoulder_angle_velocity_vs_right_knee_angle_velocity_mean_correlation**	**1.35E+01**	**2.74E-04**
**F84**	**right_shoulder_angle_velocity_vs_left_elbow_angle_velocity_mean_correlation**	**2.04E+01**	**8.44E-06**
**F85**	**right_shoulder_angle_velocity_vs_right_elbow_angle_velocity_mean_correlation**	**5.48E+00**	**1.97E-02**
**F86**	**right_shoulder_angle_velocity_vs_left_hip_angle_velocity_mean_correlation**	**8.44E+00**	**3.89E-03**
**F87**	**right_shoulder_angle_velocity_vs_right_hip_angle_velocity_mean_correlation**	**2.06E+01**	**7.66E-06**
**F88**	**right_shoulder_angle_velocity_vs_left_knee_angle_velocity_mean_correlation**	**1.70E+01**	**4.64E-05**

**F89**	**right_shoulder_angle_velocity_vs_right_knee_angle_velocity_mean_correlation**	**3.36E+01**	**1.46E-08**
**F90**	**left_elbow_angle_velocity_vs_right_elbow_angle_velocity_mean_correlation**	**8.49E+00**	**3.79E-03**
**F91**	**left_elbow_angle_velocity_vs_left_hip_angle_velocity_mean_correlation**	**1.54E+01**	**1.04E-04**
**F92**	**left_elbow_angle_velocity_vs_right_hip_angle_velocity_mean_correlation**	**1.15E+01**	**7.82E-04**
**F93**	**left_elbow_angle_velocity_vs_left_knee_angle_velocity_mean_correlation**	**2.54E+01**	**7.34E-07**
**F94**	**left_elbow_angle_velocity_vs_right_knee_angle_velocity_mean_correlation**	**1.37E+01**	**2.50E-04**
F95	right_elbow_angle_velocity_vs_left_hip_angle_velocity_mean_correlation	2.71E+00	1.01E-01
**F96**	**right_elbow_angle_velocity_vs_right_hip_angle_velocity_mean_correlation**	**2.24E+01**	**3.12E-06**
F97	right_elbow_angle_velocity_vs_left_knee_angle_velocity_mean_correlation	1.06E+00	3.03E-01
**F98**	**right_elbow_angle_velocity_vs_right_knee_angle_velocity_mean_correlation**	**7.88E+00**	**5.27E-03**
F99	left_hip_angle_velocity_vs_right_hip_angle_velocity_mean_correlation	1.22E+00	2.71E-01
**F100**	**left_hip_angle_velocity_vs_left_knee_angle_velocity_mean_correlation**	**4.62E+00**	**3.23E-02**
F101	left_hip_angle_velocity_vs_right_knee_angle_velocity_mean_correlation	4.12E-01	5.21E-01
F102	right_hip_angle_velocity_vs_left_knee_angle_velocity_mean_correlation	4.78E-04	9.83E-01
F103	right_hip_angle_velocity_vs_right_knee_angle_velocity_mean_correlation	4.22E-01	5.16E-01
F104	left_knee_angle_velocity_vs_right_knee_angle_velocity_mean_correlation	1.76E-01	6.75E-01
F105	left_shoulder_angle_acceleration_vs_right_shoulder_angle_acceleration_mean_correlation	3.84E+00	5.08E-02
**F106**	**left_shoulder_angle_acceleration_vs_left_elbow_angle_acceleration_mean_correlation**	**1.27E+01**	**4.15E-04**
F107	left_shoulder_angle_acceleration_vs_right_elbow_angle_acceleration_mean_correlation	4.36E-01	5.09E-01

F108	left_shoulder_angle_acceleration_vs_left_hip_angle_acceleration_mean_correlation	5.07E-01	4.77E-01
**F109**	**left_shoulder_angle_acceleration_vs_right_hip_angle_acceleration_mean_correlation**	**2.04E+01**	**8.69E-06**
F110	left_shoulder_angle_acceleration_vs_left_knee_angle_acceleration_mean_correlation	2.66E+00	1.04E-01
**F111**	**left_shoulder_angle_acceleration_vs_right_knee_angle_acceleration_mean_correlation**	**3.03E+01**	**7.01E-08**
**F112**	**right_shoulder_angle_acceleration_vs_left_elbow_angle_acceleration_mean_correlation**	**2.54E+01**	**7.34E-07**
F113	right_shoulder_angle_acceleration_vs_right_elbow_angle_acceleration_mean_correlation	2.62E+00	1.07E-01
**F114**	**right_shoulder_angle_acceleration_vs_left_hip_angle_acceleration_mean_correlation**	**5.77E+00**	**1.68E-02**
**F115**	**right_shoulder_angle_acceleration_vs_right_hip_angle_acceleration_mean_correlation**	**8.71E+00**	**3.38E-03**
**F116**	**right_shoulder_angle_acceleration_vs_left_knee_angle_acceleration_mean_correlation**	**1.19E+01**	**6.35E-04**
**F117**	**right_shoulder_angle_acceleration_vs_right_knee_angle_acceleration_mean_correlation**	**2.21E+01**	**3.65E-06**
**F118**	**left_elbow_angle_acceleration_vs_right_elbow_angle_acceleration_mean_correlation**	**9.51E+00**	**2.20E-03**
**F119**	**left_elbow_angle_acceleration_vs_left_hip_angle_acceleration_mean_correlation**	**7.64E+00**	**5.99E-03**
F120	left_elbow_angle_acceleration_vs_right_hip_angle_acceleration_mean_correlation	9.04E-03	9.24E-01
**F121**	**left_elbow_angle_acceleration_vs_left_knee_angle_acceleration_mean_correlation**	**1.34E+01**	**2.93E-04**
F122	left_elbow_angle_acceleration_vs_right_knee_angle_acceleration_mean_correlation	2.23E-01	6.37E-01
**F123**	**right_elbow_angle_acceleration_vs_left_hip_angle_acceleration_mean_correlation**	**9.86E+00**	**1.83E-03**
**F124**	**right_elbow_angle_acceleration_vs_right_hip_angle_acceleration_mean_correlation**	**1.84E+01**	**2.26E-05**
**F125**	**right_elbow_angle_acceleration_vs_left_knee_angle_acceleration_mean_correlation**	**7.96E+00**	**5.04E-03**
**F126**	**right_elbow_angle_acceleration_vs_right_knee_angle_acceleration_mean_correlation**	**1.03E+01**	**1.46E-03**

**F127**	**left_hip_angle_acceleration_vs_right_hip_angle_acceleration_mean_correlation**	**7.97E+00**	**5.03E-03**
**F128**	**left_hip_angle_acceleration_vs_left_knee_angle_acceleration_mean_correlation**	**4.09E+00**	**4.38E-02**
F129	left_hip_angle_acceleration_vs_right_knee_angle_acceleration_mean_correlation	2.11E+00	1.48E-01
**F130**	**right_hip_angle_acceleration_vs_left_knee_angle_acceleration_mean_correlation**	**3.90E+00**	**4.90E-02**
F131	right_hip_angle_acceleration_vs_right_knee_angle_acceleration_mean_correlation	2.80E-01	5.97E-01
F132	left_knee_angle_acceleration_vs_right_knee_angle_acceleration_mean_correlation	3.72E+00	5.47E-02
F133	left_shoulder_angle_velocity_vs_right_shoulder_angle_velocity_max_correlation	2.25E+00	1.34E-01
F134	left_shoulder_angle_velocity_vs_left_hip_angle_velocity_max_correlation	2.41E+00	1.22E-01
F135	left_shoulder_angle_velocity_vs_left_elbow_angle_velocity_max_correlation	1.84E+00	1.76E-01
F136	left_shoulder_angle_velocity_vs_right_elbow_angle_velocity_max_correlation	7.95E-02	7.78E-01
F137	left_shoulder_angle_velocity_vs_right_hip_angle_velocity_max_correlation	2.33E+00	1.28E-01
**F138**	**left_shoulder_angle_velocity_vs_left_knee_angle_velocity_max_correlation**	**5.57E+00**	**1.88E-02**
**F139**	**left_shoulder_angle_velocity_vs_right_knee_angle_velocity_max_correlation**	**1.88E+01**	**1.89E-05**
**F140**	**right_shoulder_angle_velocity_vs_left_elbow_angle_velocity_max_correlation**	**9.77E+00**	**1.92E-03**
F141	right_shoulder_angle_velocity_vs_right_elbow_angle_velocity_max_correlation	2.83E+00	9.34E-02
**F142**	**right_shoulder_angle_velocity_vs_left_hip_angle_velocity_max_correlation**	**2.10E+01**	**6.33E-06**
**F143**	**right_shoulder_angle_velocity_vs_right_hip_angle_velocity_max_correlation**	**4.11E+00**	**4.34E-02**
**F144**	**right_shoulder_angle_velocity_vs_left_knee_angle_velocity_max_correlation**	**6.99E+00**	**8.55E-03**
**F145**	**right_shoulder_angle_velocity_vs_right_knee_angle_velocity_max_correlation**	**3.65E+01**	**3.82E-09**

F146	left_elbow_angle_velocity_vs_right_elbow_angle_velocity_max_correlation	3.60E+00	5.85E-02
**F147**	**left_elbow_angle_velocity_vs_left_hip_angle_velocity_max_correlation**	**5.12E+00**	**2.42E-02**
**F148**	**left_elbow_angle_velocity_vs_right_hip_angle_velocity_max_correlation**	**4.90E+00**	**2.74E-02**
**F149**	**left_elbow_angle_velocity_vs_left_knee_angle_velocity_max_correlation**	**1.97E+01**	**1.20E-05**
**F150**	**left_elbow_angle_velocity_vs_right_knee_angle_velocity_max_correlation**	**1.64E+01**	**6.41E-05**
F151	right_elbow_angle_velocity_vs_left_hip_angle_velocity_max_correlation	8.17E-01	3.67E-01
**F152**	**right_elbow_angle_velocity_vs_right_hip_angle_velocity_max_correlation**	**1.41E+01**	**1.98E-04**
F153	right_elbow_angle_velocity_vs_left_knee_angle_velocity_max_correlation	1.91E-01	6.62E-01
F154	right_elbow_angle_velocity_vs_right_knee_angle_velocity_max_correlation	1.56E+00	2.13E-01
F155	left_hip_angle_velocity_vs_right_hip_angle_velocity_max_correlation	9.69E-01	3.25E-01
**F156**	**left_hip_angle_velocity_vs_left_knee_angle_velocity_max_correlation**	**1.06E+01**	**1.22E-03**
F157	left_hip_angle_velocity_vs_right_knee_angle_velocity_max_correlation	3.46E+00	6.37E-02
F158	right_hip_angle_velocity_vs_left_knee_angle_velocity_max_correlation	3.25E+00	7.22E-02
**F159**	**right_hip_angle_velocity_vs_right_knee_angle_velocity_max_correlation**	**8.21E+00**	**4.41E-03**
F160	left_knee_angle_velocity_vs_right_knee_angle_velocity_max_correlation	2.31E+00	1.29E-01
F161	left_shoulder_angle_acceleration_vs_right_shoulder_angle_acceleration_max_correlation	3.32E+00	6.91E-02
F162	left_shoulder_angle_acceleration_vs_left_elbow_angle_acceleration_max_correlation	8.21E-02	7.75E-01
**F163**	**left_shoulder_angle_acceleration_vs_right_elbow_angle_acceleration_max_correlation**	**8.70E+00**	**3.40E-03**
**F164**	**left_shoulder_angle_acceleration_vs_left_hip_angle_acceleration_max_correlation**	**7.01E+00**	**8.45E-03**

**F165**	**left_shoulder_angle_acceleration_vs_right_hip_angle_acceleration_max_correlation**	**7.44E+00**	**6.69E-03**
F166	left_shoulder_angle_acceleration_vs_left_knee_angle_acceleration_max_correlation	3.56E-01	5.51E-01
**F167**	**left_shoulder_angle_acceleration_vs_right_knee_angle_acceleration_max_correlation**	**1.25E+01**	**4.50E-04**
**F168**	**right_shoulder_angle_acceleration_vs_left_elbow_angle_acceleration_max_correlation**	**2.18E+01**	**4.33E-06**
F169	right_shoulder_angle_acceleration_vs_right_elbow_angle_acceleration_max_correlation	1.58E+00	2.09E-01
**F170**	**right_shoulder_angle_acceleration_vs_left_hip_angle_acceleration_max_correlation**	**7.58E+00**	**6.20E-03**
F171	right_shoulder_angle_acceleration_vs_right_hip_angle_acceleration_max_correlation	3.07E+00	8.08E-02
**F172**	**right_shoulder_angle_acceleration_vs_left_knee_angle_acceleration_max_correlation**	**1.03E+01**	**1.47E-03**
**F173**	**right_shoulder_angle_acceleration_vs_right_knee_angle_acceleration_max_correlation**	**9.77E+00**	**1.92E-03**
F174	left_elbow_angle_acceleration_vs_right_elbow_angle_acceleration_max_correlation	2.61E-01	6.10E-01
F175	left_elbow_angle_acceleration_vs_left_hip_angle_acceleration_max_correlation	5.78E-03	9.39E-01
F176	left_elbow_angle_acceleration_vs_right_hip_angle_acceleration_max_correlation	8.07E-01	3.69E-01
**F177**	**left_elbow_angle_acceleration_vs_left_knee_angle_acceleration_max_correlation**	**7.14E+00**	**7.88E-03**
F178	left_elbow_angle_acceleration_vs_right_knee_angle_acceleration_max_correlation	4.45E-01	5.05E-01
F179	right_elbow_angle_acceleration_vs_left_hip_angle_acceleration_max_correlation	3.13E+00	7.76E-02
**F180**	**right_elbow_angle_acceleration_vs_right_hip_angle_acceleration_max_correlation**	**5.65E+00**	**1.80E-02**
F181	right_elbow_angle_acceleration_vs_left_knee_angle_acceleration_max_correlation	3.59E+00	5.89E-02
F182	right_elbow_angle_acceleration_vs_right_knee_angle_acceleration_max_correlation	1.82E+00	1.79E-01
**F183**	**left_hip_angle_acceleration_vs_right_hip_angle_acceleration_max_correlation**	**1.61E+01**	**7.24E-05**

**F184**	**left_hip_angle_acceleration_vs_left_knee_angle_acceleration_max_correlation**	**8.68E+00**	**3.42E-03**
F185	left_hip_angle_acceleration_vs_right_knee_angle_acceleration_max_correlation	1.77E-02	8.94E-01
**F186**	**right_hip_angle_acceleration_vs_left_knee_angle_acceleration_max_correlation**	**4.82E+00**	**2.88E-02**
F187	right_hip_angle_acceleration_vs_right_knee_angle_acceleration_max_correlation	2.31E+00	1.30E-01
F188	left_knee_angle_acceleration_vs_right_knee_angle_acceleration_max_correlation	8.44E-01	3.59E-01
**F189**	**left_wrist_velocity_vs_right_wrist_velocity_max_correlation**	**1.20E+01**	**5.89E-04**
**F190**	**left_wrist_velocity_vs_left_ankle_velocity_max_correlation**	**1.38E+01**	**2.30E-04**
F191	left_wrist_velocity_vs_right_ankle_velocity_max_correlation	3.17E+00	7.59E-02
**F192**	**right_wrist_velocity_vs_left_ankle_velocity_max_correlation**	**1.16E+01**	**7.29E-04**
**F193**	**right_wrist_velocity_vs_right_ankle_velocity_max_correlation**	**2.08E+01**	**6.96E-06**
**F194**	**left_ankle_velocity_vs_right_ankle_velocity_max_correlation**	**1.91E+01**	**1.64E-05**
**F195**	**left_wrist_acceleration_vs_right_wrist_acceleration_max_correlation**	**1.28E+01**	**3.89E-04**
F196	left_wrist_acceleration_vs_left_ankle_acceleration_max_correlation	2.51E+00	1.14E-01
**F197**	**left_wrist_acceleration_vs_right_ankle_acceleration_max_correlation**	**7.81E+00**	**5.48E-03**
**F198**	**right_wrist_acceleration_vs_left_ankle_acceleration_max_correlation**	**1.20E+01**	**5.92E-04**
**F199**	**right_wrist_acceleration_vs_right_ankle_acceleration_max_correlation**	**1.64E+01**	**6.37E-05**
**F200**	**left_ankle_acceleration_vs_right_ankle_acceleration_max_correlation**	**1.27E+01**	**4.11E-04**
F201	right_shoulder_angle_entropy	2.97E-04	9.86E-01
F202	left_shoulder_angle_entropy	1.01E+00	3.16E-01
F203	left_elbow_angle_entropy	3.22E-02	8.58E-01
F204	right_elbow_angle_entropy	2.61E+00	1.07E-01

F205	left_hip_angle_entropy	2.18E-04	9.88E-01
F206	right_hip_angle_entropy	2.21E-01	6.39E-01
**F207**	**left_knee_angle_entropy**	**6.20E+00**	**1.32E-02**
F208	right_knee_angle_entropy	1.02E-03	9.75E-01
F209	left_elbow_velocity_entropy	7.04E-01	4.02E-01
F210	right_elbow_velocity_entropy	3.99E-02	8.42E-01
F211	left_wrist_velocity_entropy	8.73E-01	3.51E-01
F212	right_wrist_velocity_entropy	3.43E+00	6.49E-02
F213	left_knee_velocity_entropy	7.00E-04	9.79E-01
**F214**	**right_knee_velocity_entropy**	**7.32E+00**	**7.14E-03**
F215	left_ankle_velocity_entropy	2.52E+00	1.13E-01
F216	right_ankle_velocity_entropy	1.01E+00	3.14E-01
**F217**	**left_elbow_acceleration_entropy**	**5.16E+00**	**2.37E-02**
F218	right_elbow_acceleration_entropy	3.64E+00	5.70E-02
F219	left_wrist_acceleration_entropy	4.31E-03	9.48E-01
**F220**	**right_wrist_acceleration_entropy**	**9.38E+00**	**2.36E-03**
F221	left_knee_acceleration_entropy	2.61E+00	1.07E-01
F222	right_knee_acceleration_entropy	2.11E+00	1.47E-01
**F223**	**left_ankle_acceleration_entropy**	**5.51E+00**	**1.95E-02**
F224	right_ankle_acceleration_entropy	4.91E-01	4.84E-01
F225	Mean_of_max_velocity_of_all_joints	7.71E-01	3.80E-01
**F226**	**mean_of_max_velocity_correlation_of_all_joints**	**3.44E+01**	**1.01E-08**
F227	mean_of_acceleration_entropy_of_all_joints	2.71E+00	1.00E-01
